# Supplementary material for: Distribution of microbial carrageenan foraging pathways reveals a widespread latent trait within the ruminant intestinal microbiome
Source: Nat Commun. 2026 May 12;17:4237. doi: 10.1038/s41467-026-70776-7 (PMC13168452; doi:10.1038/s41467-026-70776-7)
Supplement: Supplementary file 1 — Supplementary Information [file 41467_2026_70776_MOESM1_ESM.pdf]

Supplementary Figures

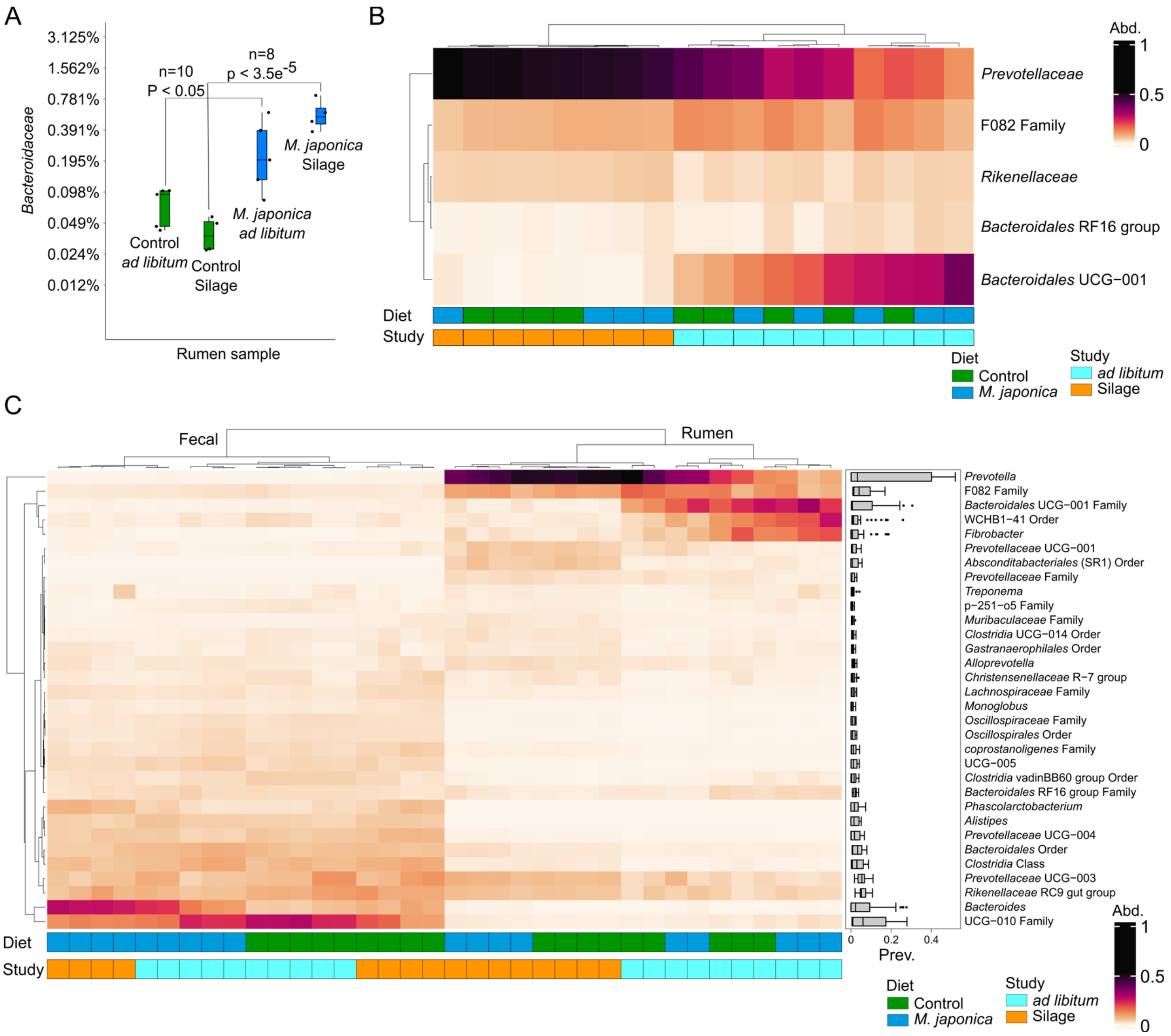

Supplementary Figure 1: *M. japonica* supplementation influences the fecal microbiome greater than the rumen microbiome. A) Percent abundance change of *Bacteroidaceae* between diet and study rumen samples. A linear regression model of % composition was generated using microViz[1], and a Tukey test was used to measure significance (adjusted p-value; two-sided)[2] **B)** 16S rRNA gene relative abundance of Genus' above 10,000 reads within rumen samples. **C)** 16S rRNA gene relative abundance of Genus' above 10,000 reads within rumen and fecal samples. Diet (Control: n=9 and *M. japonica*: n=9 supplemented) and Study (*ad libitum*: n=10 and Silage: n=8) are denoted below the heatmap. Source Data is associated with this figure.

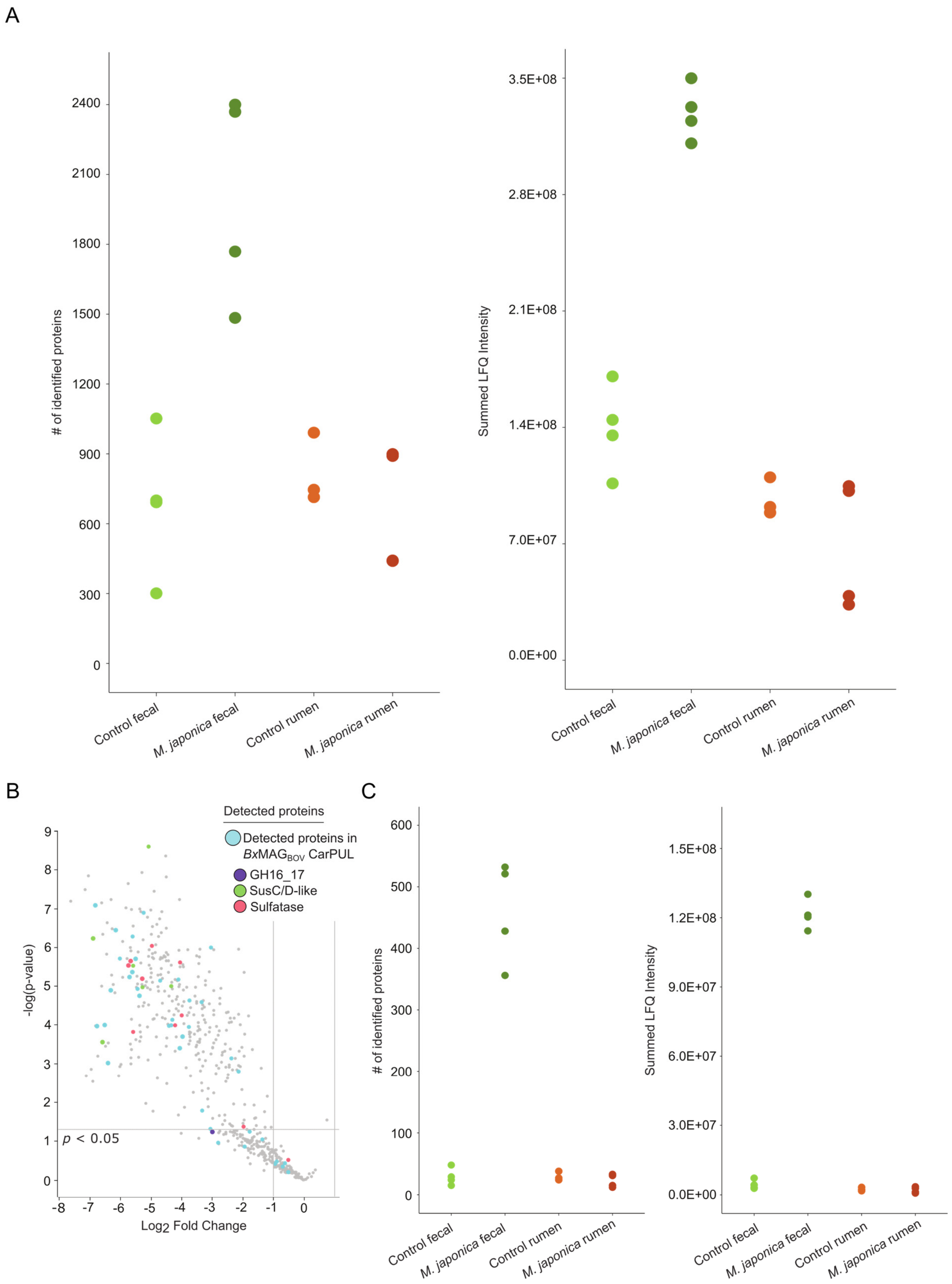

A
# of identified proteins
2400
2100
1800
1500
1200
900
600
300
0
Control fecal
M. japonica fecal
Control rumen
M. japonica rumen
Summed LFQ Intensity
3.5E+08
2.8E+08
2.1E+08
1.4E+08
7.0E+07
0.0E+00
B
Detected proteins
Detected proteins in BxMAGBOV CarPUL
GH16_17
SusC/D-like
Sulfatase
-log(p-value)
p < 0.05
Log2 Fold Change
C
600
500
400
300
200
100
1.5E+08
1.2E+08
9.0E+07
6.0E+07
3.0E+07

Supplementary Figure 2: A) Dot plots for **left:** total proteins identified, and **right:** summed metaproteomic expression, presented as summed LFQ intensity in rumen and fecal samples from cattle (n=4) fed a control diet and a silage diet supplemented with 5% *M. japonica*. Detected LFQ intensities for identified proteins can be found in Supplementary Data 3. **B)** Volcano plot showing *Bx*MAG$_{BOV}$ proteins with significantly different protein expression recovered from fecal samples from cattle fed diet with 5% inclusion level of *M. japonica* vs those fed the control diet (two-sided Student's T-test; S0=0). Difference in protein expression is displayed as Log$_2$ fold change in LFQ intensities. **C)** Dots plots above were condensed to show total proteins identified (**left**) and LFQ intensity (**right**) from only proteins within *Bx*MAG$_{BOV}$.

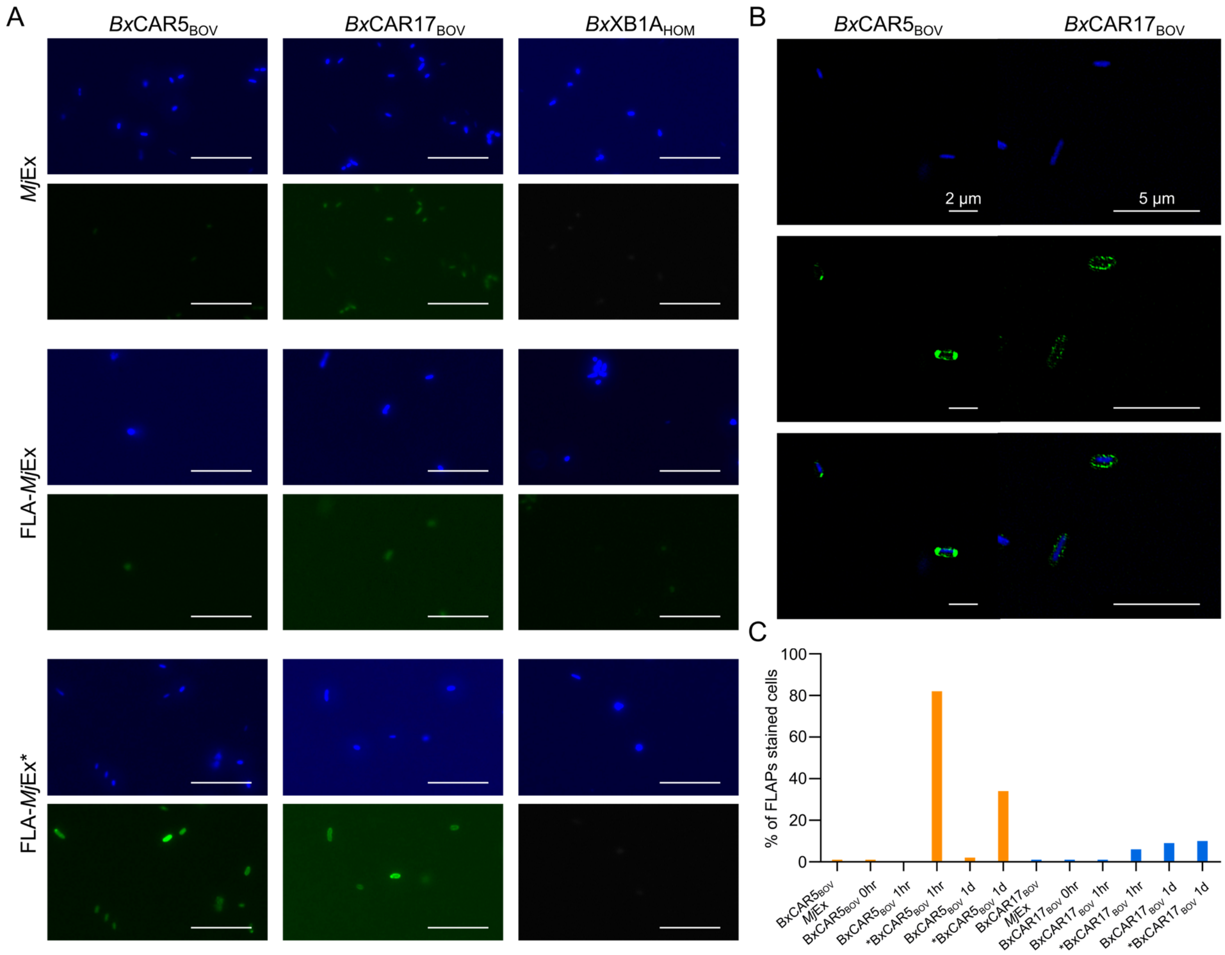

Supplementary Figure 3: Visualization of *Bacteroides* spp. interacting with fluorescently labelled *M. japonica* extract. A) *Bx*Car5$_{BOV}$, *Bx*Car17$_{BOV}$, and *Bx*XB1A$_{HOM}$, were incubated with 0.2% of unlabelled *M. japonica* extract (*Mj*Ex) and fluorescently labelled *M. japonica* extract (FLA-*Mj*Ex) for 1 day. Samples were co-stained with DAPI and imaged using an epifluorescence microscope (LED light cubes DAPI (EX: 385/30 EM: 450/50 DM: 425, ED light cubes FLA-*Mj*Ex (EX: 470/40 EM: 525/50 DM: 495). FLA-*Mj*Ex: not primed, isolate inoculum grown on galactose for 24 h (no *Mj*Ex). FLA-*Mj*Ex*: primed, isolate inoculum grown for 24 h on *Mj*Ex. Scale bar = 10 µm. **B)** Super-resolution structured illumination microscopy of *Bx*Car5$_{BOV}$ and *Bx*Car17$_{BOV}$ primed of *Mj*Ex and incubated with FLA-*Mj*Ex after 1 day. **C)** Percentage of cells within field of view stained with FLA-*Mj*Ex. Observed (auto)fluorescence within negative *Bx*XB1A$_{HOM}$ were subtracted from the values.

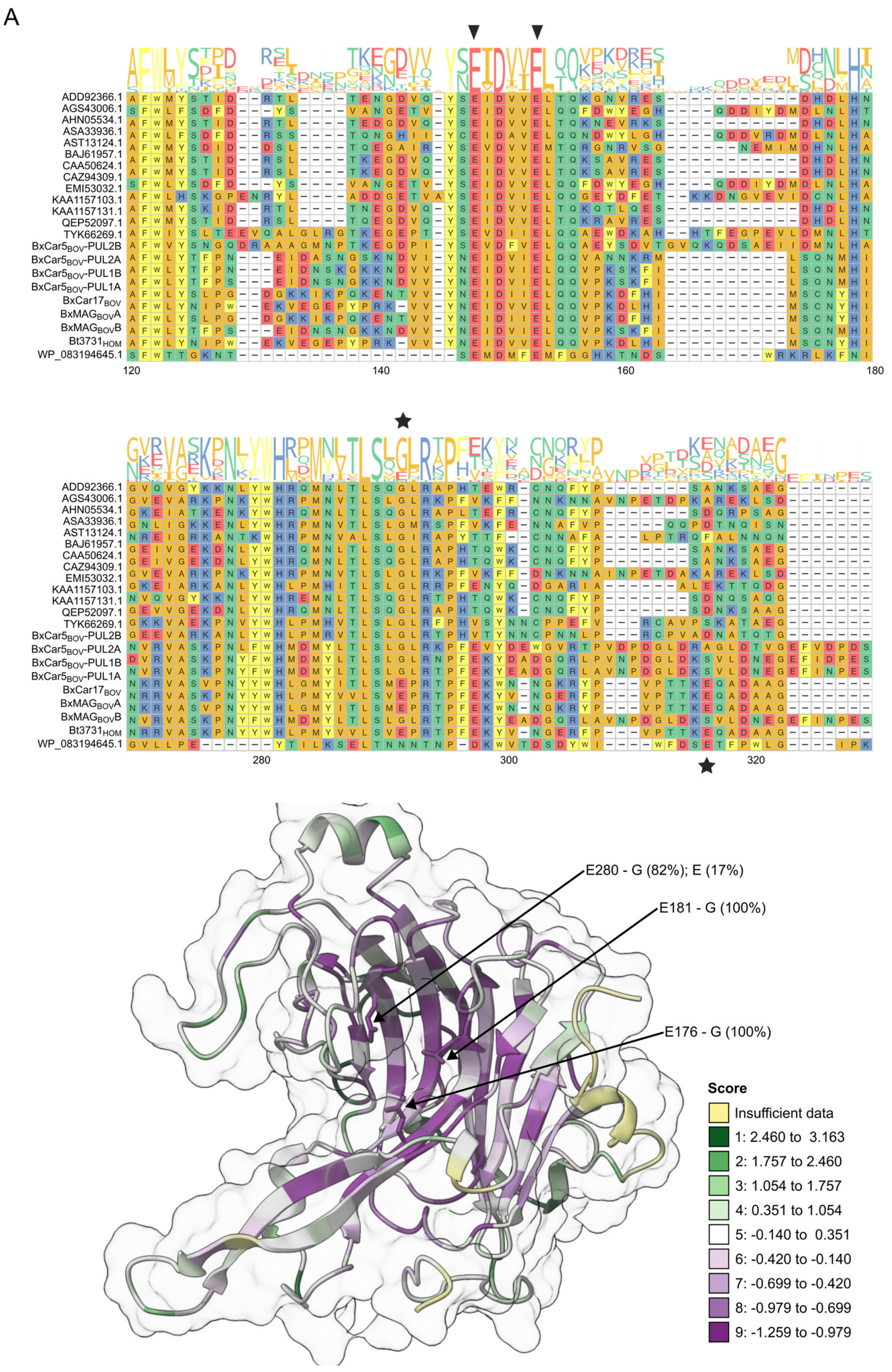
A
ADD92366.1
AGS43006.1
AHN05534.1
ASA33936.1
AST13124.1
BAJ61957.1
CAA50624.1
CAZ94309.1
EMI53032.1
KAA1157103.1
KAA1157131.1
QEP52097.1
TYK66269.1
BxCar5BOV-PUL2B
BxCar5BOV-PUL2A
BxCar5BOV-PUL1B
BxCar5BOV-PUL1A
BxCar17BOV
BxMAGBOVA
BxMAGBOVB
Bt3731HOM
WP_083194645.1
120
140
160
180
280
300
320
B
E280 - G (82%); E (17%)
E181 - G (100%)
E176 - G (100%)
Score
Insufficient data
1: 2.460 to 3.163
2: 1.757 to 2.460
3: 1.054 to 1.757
4: 0.351 to 1.054
5: -0.140 to 0.351
6: -0.420 to -0.140
7: -0.699 to -0.420
8: -0.979 to -0.699
9: -1.259 to -0.979

Supplementary Figure 4: Sequence alignment of GH16_17 members. A) Sequence alignment of characterized GH16_17 members, and *Bx*CarPUL$_{BOV}$ GH16s. GH16_13 member (WP_083194645.1; Cgbk16A_Wf) was added as a outgroup. Catalytic acid and nucleophile glutamic acids (E) are denoted with black triangles. The black star denotes the amino acid, glycine – G or glutamic acid – E, which allows for or occludes 4S-modified anhydro-galactose in the -1 subsite, respectively. **B)** ConSurf[3] analysis of *Bx*MAG$_{BOV}$ GH16_17A. 92 sequences were aligned with *Bx*MAG$_{BOV}$ using default settings with the color score present in the legend. Critical active site residues are labelled with the percent abundance of amino acid variety in that position. The active site residue occluding or allowing for 4S-modified anhydro-galactose is labelled E280. Source Data is associated with this figure.

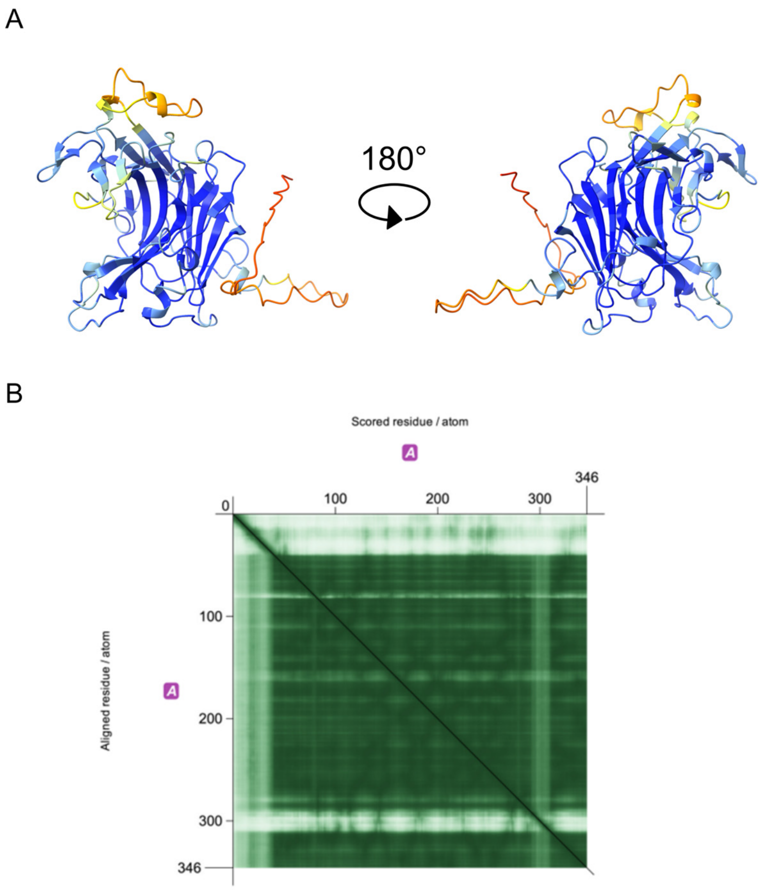

Supplementary Figure 5: AlphaFold3 confidence values of *Bx*MAG GH16B. A) pLDDT score overlaid from two viewing angles rotated 180° across the Y-axis. **B)** The predicted aligned error (PAE) of *Bx*MAG GH16B.

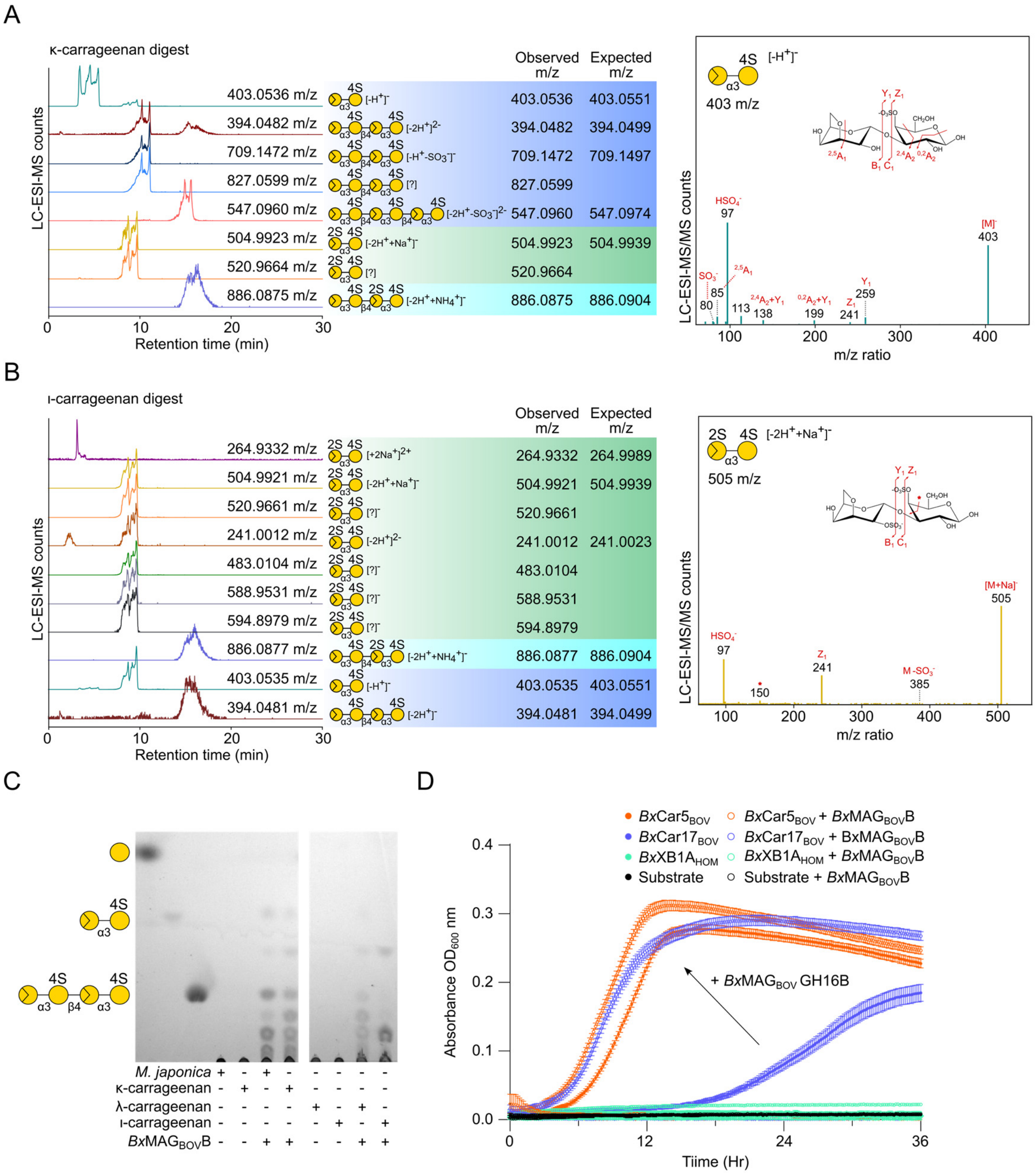
A
κ-carrageenan digest
LC-ESI-MS counts
403.0536 m/z
394.0482 m/z
709.1472 m/z
827.0599 m/z
547.0960 m/z
504.9923 m/z
520.9664 m/z
886.0875 m/z
Retention time (min)
Observed m/z
Expected m/z
403.0536 403.0551
394.0482 394.0499
709.1472 709.1497
827.0599
547.0960 547.0974
504.9923 504.9939
520.9664
886.0875 886.0904
403 m/z
LC-ESI-MS/MS counts
m/z ratio
B
ι-carrageenan digest
264.9332 m/z
504.9921 m/z
520.9661 m/z
241.0012 m/z
483.0104 m/z
588.9531 m/z
594.8979 m/z
886.0877 m/z
403.0535 m/z
394.0481 m/z
264.9332 264.9989
504.9921 504.9939
520.9661
241.0012 241.0023
483.0104
588.9531
594.8979
886.0877 886.0904
403.0535 403.0551
394.0481 394.0499
505 m/z
C
M. japonica
κ-carrageenan
λ-carrageenan
ι-carrageenan
BxMAGBOVB
D
BxCar5BOV
BxCar17BOV
BxXB1AHOM
Substrate
BxCar5BOV + BxMAGBOVB
BxCar17BOV + BxMAGBOVB
BxXB1AHOM + BxMAGBOVB
Substrate + BxMAGBOVB
+ BxMAGBOV GH16B
Absorbance OD600 nm
Tiime (Hr)

Supplementary Figure 6: Analysis of *M. japonica*, κ-carrageenan, and ι-carrageenan enzyme digests. Carrageenan substrates were incubated with *Bx*MAG$_{BOV}$ GH16B enzyme and analyzed by LC-ESI-MS. Extracted ion chromatograms are shown locally normalized for **A)** κ-carrageenan and **B)** ι-carrageenan digests. ESI-MS/MS with HCD was performed in order to identify the extracted ions, and MS2 product ion spectra are shown with the carbohydrate fragmentation depicted and labeled. The identified ions correspond to neo-κ-carrabiose **C)** and a neo-ι-carrabiose **D)** as majority enzymatic products from their respective digests. **E)** TLC of commercially available carrageenans and *Mj*Ex digested with *Bx*MAG$_{BOV}$ GH16_17B. Products compared to galactose, neo-κ-carrabiose, and neo-κ-carratetraose standards. Monosaccharide symbols are displayed according to the Symbol Nomenclature for Glycans system (1). TLCs were run a minimum of 3x; blots were processed in parallel. **F)** Growth cultures of *Bx*Car isolates on 0.3% *M. japonica* supplemented with 1 mM *Bx*MAG$_{BOV}$ GH16_17B. OD_{600} nm was observed every 10 minutes. Negative ODs were excluded from the plot. Error bars indicate minimum and maximum OD between 3 replicates (n=3).

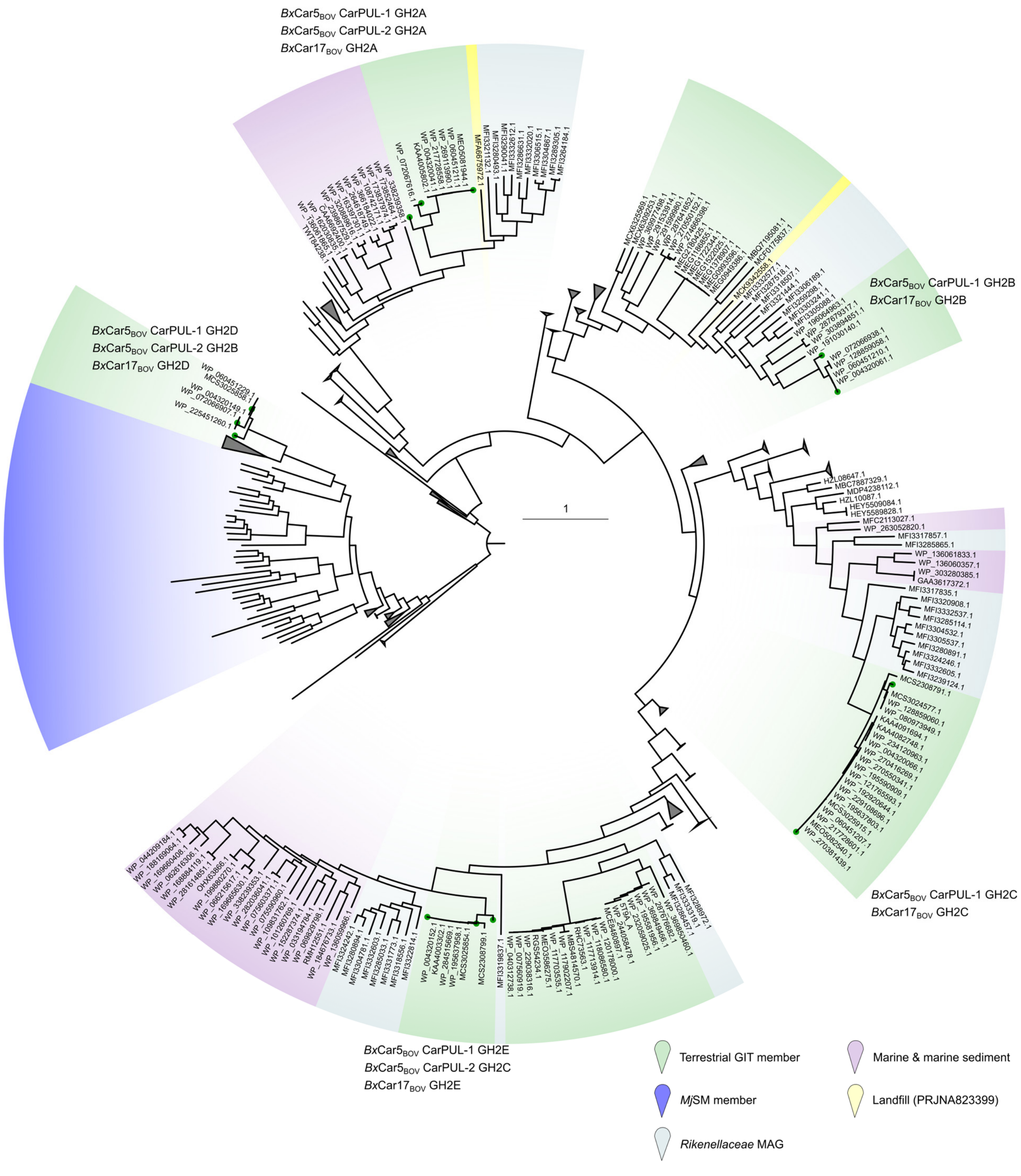

*Bx*Car5$_{BOV}$ CarPUL-1 GH2A
*Bx*Car5$_{BOV}$ CarPUL-2 GH2A
*Bx*Car17$_{BOV}$ GH2A
*Bx*Car5$_{BOV}$ CarPUL-1 GH2B
*Bx*Car17$_{BOV}$ GH2B
*Bx*Car5$_{BOV}$ CarPUL-1 GH2D
*Bx*Car5$_{BOV}$ CarPUL-2 GH2B
*Bx*Car17$_{BOV}$ GH2D
1
*Bx*Car5$_{BOV}$ CarPUL-1 GH2C
*Bx*Car17$_{BOV}$ GH2C
*Bx*Car5$_{BOV}$ CarPUL-1 GH2E
*Bx*Car5$_{BOV}$ CarPUL-2 GH2C
*Bx*Car17$_{BOV}$ GH2E
Terrestrial GIT member
Marine & marine sediment
*Mj*SM member
Landfill (PRJNA823399)
Rikenellaceae MAG

Supplementary Figure 7: Homology of GH2s from CarPULs bacteria in the NCBI database. SACCHARIS v2 phylogenies were generated between the top 100 BLAST hits towards *Bx*CarBOV CarPUL GH2 members, characterized GH2 members from the CAZy database, predicted GH2 members from *Mj*SM metagenomes, and *Bx*CarBOV CarPUL GH2s. Clades are colored based on their host environment; Green: terrestrial vertebrate *Bacteroides* spp., blue: *Mj*SM members, teal: *K. sydneyanus* GIT members, purple: marine/sediment microorganisms, and yellow: landfill. Individual nodes are denoted by a pin, and CarPUL members are denoted with labelling and green dots at the node. Clades absent of CarPUL members were collapsed for phylogeny clarity. Source Data is associated with this figure.

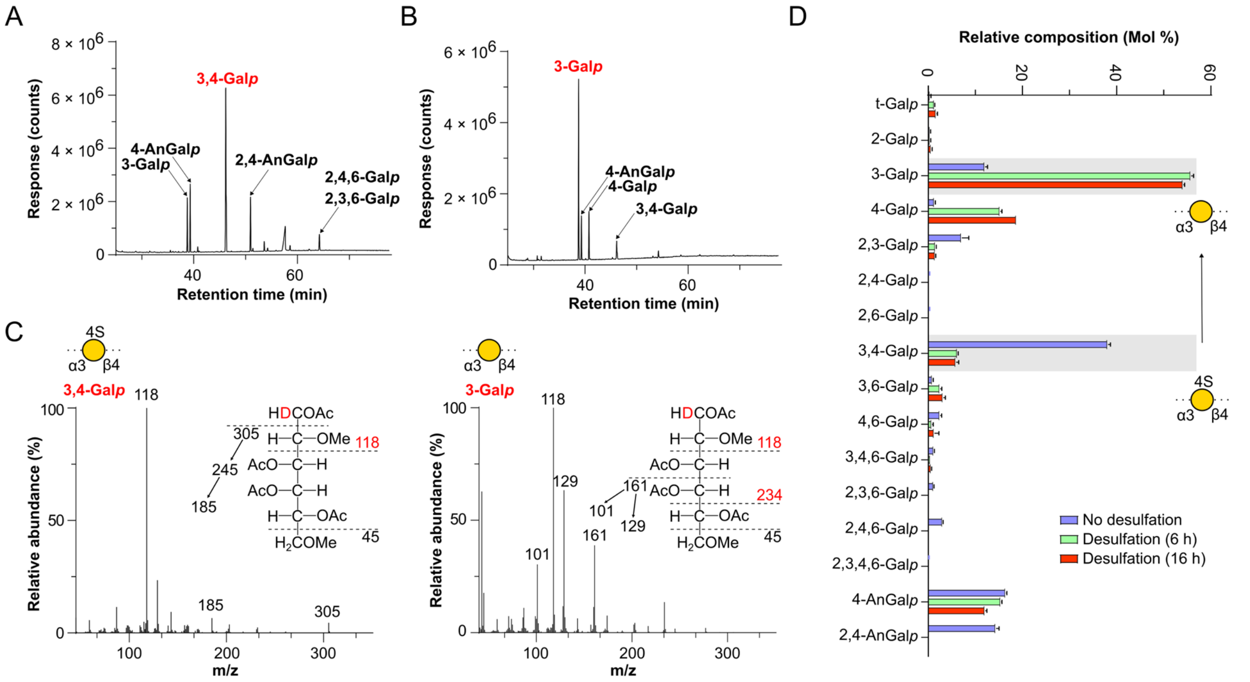

Supplementary figure 8: Glycosidic linkage analysis (methylation-GC-MS analysis) of *M. japonica* galactan and its desulfation product. GC-TIC chromatograms of PMAAs from destarched, water-soluble *M. japonica* extract (*Mj*Ex) **A)** before solvolytic desulfation and **B)** after 16 h of solvolytic desulfation. **C)** EI-MS spectra and ion fragmentation patterns of PMAAs from the largest peaks within GC-TIC chromatograms. Top: no desulfation (3,4-Galp). Bottom: 16 h desulfation (3-Galp). **D)** Comparison of relative composition (Mol%) of observed PMAAs from samples with no desulfation and those subjected to 6 h and16 h of desulfation. Source Data is associated with this figure.

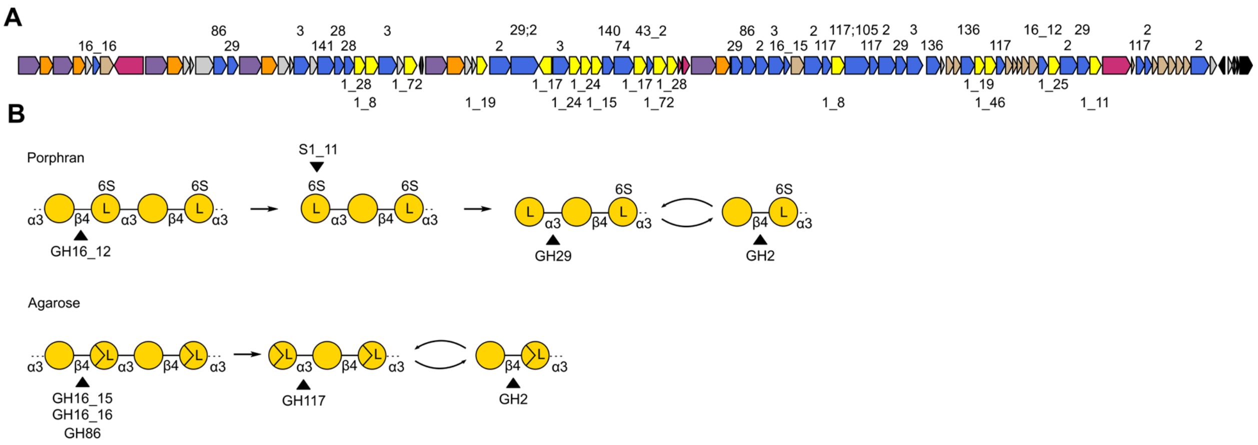

Supplementary Figure 9: Porphyran/agarose PUL identified within the giraffe metagenome. A) PUL diagram of the porphyran/agarose PUL within *Bz*Car$_{GIR}$. B) Predicted depolymerization pathway of porphyran and agarose in *Bz*Car$_{GIR}$.

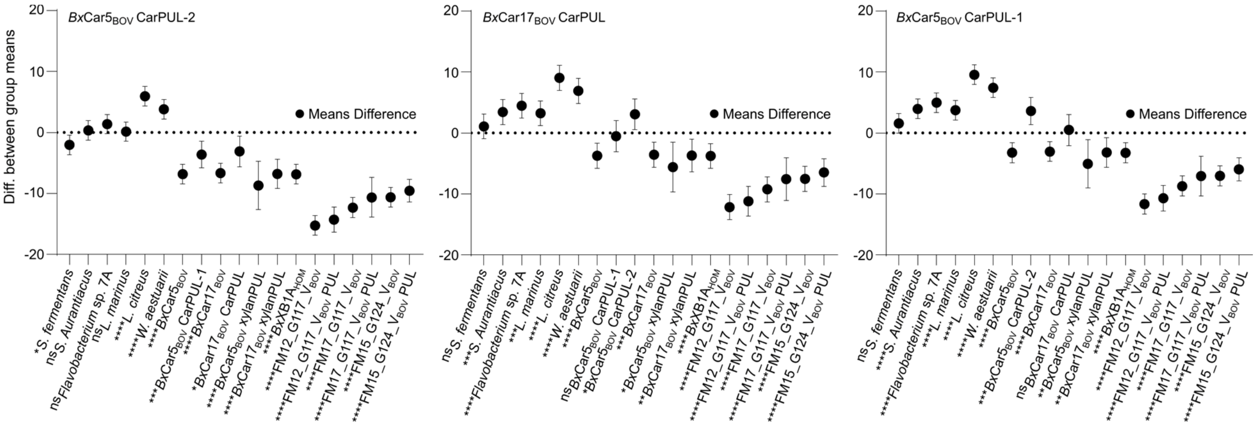

Supplementary figure 10: Mean percent GC content compared between *Bx*Car CarPULs and *Bx*Car genomes is significantly different. Mean GC% content of CarPULs was compared to that of their genome (whole genome and terrestrial xylan PULs[4] within their genome), marine pelagic and sediment microbiota gene GC% content, and gene content of 3 *R. alistipes* MAGs isolated from the GIT of *K. sydneyanus*. From left to right: *Bx*Car5$_{BOV}$ PUL-2, *Bx*Car17$_{BOV}$ CarPUL*, Bx*Car5$_{BOV}$ PUL-1. Mean %GC abundance between *Bx*Car PULs and control and marine datasets were compared using Graphpad prism. One-Way Anova with Games-Howell multiple comparisons were calculated against each PUL to identify the difference between the means (Supplementary Data 11). Along with previously stated controls, a native xylan PUL from each isolate was used. ns: $P > 0.05$; *: $P \leq 0.05$; **: $P \leq 0.01$; ***: $P \leq 0.001$; ****: $P \leq 0.0001$. Source Data is associated with this figure.

Supplementary Tables

Supplementary Table 1: Bovine diets used within this study.

Ingredient	AF kgs	DM kgs
Mineral	0.1	0.1
Whey	23	1.5
Grass Hat	2	1.74
Oat Hulls	1	0.75
Tofu Blend	3	1.27
Hydrogreen	14.5	3.34
3rd Cut Grass Silage	5.75	2.67
*Mazzaella japonica**	0.13	0.11

*Included in *M. japonica* silage diet

Supplementary Table 2: Parameters for ESI-MSn on the Orbitrap Fusion Tribrid.

Parameter (units)	Value
ESI	
Spray voltage: negative ion (V)	2500
Sheath Gas (Arb)	45
Aux Gas (Arb)	10
Sweep Gas (Arb)	1
Ion Transfer Tube Temp (°C)	325
Vaporizer Temp (°C)	200
MS	
Detector Type	Orbitrap
Orbitrap Resolution	120K
Mass Range	Normal
Scan Range (m/z)	150-2000
RF Lens (%)	50
MS2	
Collision Energy Type	Normalized
Isolation Mode	Quadrupole
Activation Type	HCD
Collision Energy Mode	Stepped
Collision Energies (%)	30,45,60,80
Detector Type	Orbitrap
Orbitrap Resolution	30K

Supplementary Table 3: Data collection and refinement statistics of *Bx*MAG$_{BOV}$ GH16_17A.

Parameter (units)	BxMAG$_{Bov}$A
Data Collection	
Beamline	CMCF-ID (CLS)
Wavelength	0.95372
Space Group	$P22_12_1$
a, b, c (Å)	56.72, 69.63, 109.06
a, b, g (º)	90, 90, 90
Resolution (Å)	30.00-2.00 (2.05-2.00)
R_{meas}	0.162 (1.140)
R_{pim}	0.071 (0.493)
CC1/2	0.994 (0.586)
<I/sI>	7.5 (1.6)
Completeness (%)	99.5 (99.6)
Redundancy	4.7 (4.9)
No. of Reflections	139297 (10590)
No. Unique	29675 (2176)
Refinement	
Resolution (Å)	2
Reflections used in refinement	29642 (2644)
Reflections used for R-free	1473 (130)
R_{work}	0.175 (0.271)
R_{free}	0.212 (0.320)
**No. of Atoms**	
Protein	2562 (310 residues)
Non-hydrogen atoms	2873
Ligands	43
Water	268
***B*-factors**	
Average B-factor	32.55
Protein	31.71

Ligand	43.47
Water	38.85
RMS Bond Lengths (Å)	0.007
RMS Bond Angles (°)	0.896
Ramachandran Preferred (%)	95.5
Ramachandran Allowed (%)	4.5
Ramachandran Disallowed (%)	0
Rotamer outliers (%)	0
Clashscore	2.36
PDB ID	9EFL

Supplementary Notes

Supplementary Note 1

Sulfated galactans were extracted from *M. japonica* and studied by linkage analysis via methylation-GC-MS analysis of PMAAs prepared from the native and desulfated products[5, 6]. Methylation-GC of the sulfated galactan without desulfation showed a high level of 3,4-Gal*p* at 38.1%, followed by 4-AnGal*p* (16.5 %), 2,4-AnGal*p* (14.4 %), 3-Gal*p* (12.1 %), 2,3-Gal*p* (7.1 %), 2,4,6-Gal*p* (3.1 %), 4,6-Gal*p* (2.5 %) (Supplementary Fig. 8A), and various other minor Gal*p* linkages (Source Data). Compared to the sample without desulfation, the sample subjected to 6 h of solvolytic desulfation[7] was dominated by 3-Gal*p* (55.8 %), followed by 4-AnGal*p* (15.5 %), 4-Gal*p* (15.3 %), 3,4-Gal*p* (6.2 %), 3,6-Gal*p* (2.5 %), and other minor Gal*p*. This was not appreciably different than desulfation at 16 h (Supplementary Fig. 8B). Glycosidic linkages of galactoses were confirmed based on the EI-MS ion fragmentation patterns of their PMAAs (Supplementary Fig. 8C), with reference to published patterns in the literature[8]. The results suggested that the majority of 3,4-Gal*p* was converted to 3-Gal*p* due to desulfation at the *O*-4 position (Supplementary Fig. 8D). A considerable amount of 4-Gal*p* was also detected in the desulfated galactan, indicating that the 4-linked galactose residues were sulfated as well. There was no 2,4-AnGal*p* was detected in the desulfated samples, indicating the complete conversion of 2,4-AnGal*p* to 4-AnGal*p* by desulfation at the *O*-2 position. The total level of AnGal in the desulfated sample was less than half of that in the untreated sample, indicating partial degradation of AnGal caused by solvolytic desulfation[7]. This was further supported by the lower level of anhydro sugar in the 16 h treatment compared to the 6 h one.

Supplementary Note 2

Mean GC content percentage between the *Bx*Car$_{BOV}$ genomes and CarPULs was compared to that of control *Bx*XB1A$_{HOM}$, xylan PULs (terrestrial plant polysaccharide) from *Bx*Car5$_{BOV}$ and 17, and marine/sediment microorganisms: *L. citreus* and *S fermentans, L. marinus, Saccharicrinis aurantiacus, Flavobacterium sp. 7A,* and *Wenyingzhuangia aestuarii*. These were selected based on the high homology observed in CarPUL CAZyme BLAST results. The mean GC% from *Bx*CAR$_{BOV}$ CarPUL genes was more similar to those of marine pelagic and sediment organisms genes than to the remainder of the BxCAR$_{BOV}$ genome (Supplementary Fig. 9). Through Games Howell's multiple comparisons tests, the mean GC% of *Bx*Car5$_{BOV}$ CarPUL-1 (39.3 ± 4.8) was more closely aligned with the *S. fermentans* genome (37.7 ± 3.8^{ns}) than to itself (42.5 ± 5.3****), a terrestrial xylan PUL[4] within the *Bx*Car5$_{BOV}$ genome (44.4 ± 5.0**), and the reference *Bx*XB1A$_{HOM}$ genome (42.6 ± 5.0****); this pattern is repeated between the *Bx*Car17$_{BOV}$ CarPUL and similar datasets. *Bx*Car5$_{BOV}$ CarPUL-2 (35.7 ± 3.6) showed the highest similarity towards marine pelagic and sediment organisms, with GC% values showing no significant difference to ORFs within *S. aurantiacus* (35.4 ± 3.4^{ns}), *Flavobacterium* sp. 7A (34.3 ± 3.6^{ns}), and *L. marinus* (35.6 ± 3.1^{ns}). All GC values and statistics are in Source Data.

Supplementary Methods

Supplementary Method 1

Ball-milled dry powder of *M. japonica* (137.6 g) was soaked in 1.2 L of hexane under magnetic stirring for 2 h in a 2 L glass beaker covered with aluminum foil to reduce evaporation. The mixture was left undisturbed overnight to allow the powder to settle. The supernatant was carefully decanted using a glass pipette, leaving approximately 1 cm of liquid above the interface to avoid disturbing the precipitate. An additional 1.2 L of hexane was added, and the extraction process was repeated. The precipitate was then resuspended in 1.2 L of 95% (v/v) ethanol, magnetically stirred for 8 h, and centrifuged at 3,000 × *g* for 30 min at room temperature. The resulting pellet was resuspended in 1.2 L of 80% (v/v) ethanol, with the same stirring and centrifugation conditions applied, and the extraction process was repeated. The resulting pellet was evaporated to dryness in 50 mL centrifuge tubes using SpeedVac (Thermo Fisher Scientific, MA, USA). The dried

sample was then transferred to a glass beaker and underwent two rounds of water extraction at room temperature with constant magnetic stirring, followed by three rounds of hot water extraction at 70 °C in an incubator. Each extraction used 2 L of deionized water and lasted 8 h, with the beaker covered with aluminum foil. After each extraction, centrifugation (3,000 × *g*, 30 min, room temperature) was conducted, and the supernatant was collected while the pellet was carried forward to the next extraction. Supernatants from all the water extractions were pooled, poured into 40 L of absolute ethanol, and left at room temperature overnight, followed by centrifugation (3,000 × *g*, 30 min, room temperature). The precipitate was evaporated to dryness in 50 mL centrifuge tubes using the SpeedVac, redissolved in 2 L of deionized water by incubating at 70 °C overnight, and freeze-dried (56.7 g).

Dry crude polysaccharide (14.4 g) was dissolved in 1 L of deionized water by incubating at 70 °C overnight. After that, 2 mL of thermostable α-amylase (3,000 units/mL, Megazyme, Ireland) was added, and the mixture was incubated at 70 °C for 8 h [9]. The solution was then poured into 4 L of absolute ethanol, left standing at 4 °C overnight, and centrifuged (3,000 × *g*, 30 min, room temperature). The residue was evaporated to dryness using the SpeedVac, redissolved in 500 mL of deionized water by incubating at 70 °C overnight, extensively dialyzed with molecular weight cut-off (MWCO) of 6,000-8,000 Da against deionized water at 4 °C, and freeze-dried (13.8 g).

Amylase-treated polysaccharide (915 mg) was dissolved in 400 mL of deionized water by incubating at 70 °C overnight. The resulting solution was cooled to room temperature, left standing at 4 °C overnight, and then centrifuged (3,000 × *g*, 30 min, room temperature). The supernatant was vigorously stirred magnetically to create a water tunnel in a 2 L glass beaker. Absolute ethanol was slowly added dropwise to achieve a 15% (w/w) ethanol concentration. The mixture was kept at 4 °C for 8 h, followed by centrifugation (3,000 × *g*, 30 min, room temperature). Absolute ethanol was then added dropwise to the vigorously stirred supernatant until the ethanol concentration reached 30% (w/w), followed by standing at 4 °C and centrifugation as described above. The resulting supernatant underwent two additional cycles of ethanol precipitation, with ethanol concentrations gradually increased to 45% and 60% (w/w), respectively. The final supernatant was evaporated to dryness in 50 mL centrifuge tubes using the SpeedVac. The dry sample was redissolved in 100 mL of deionized water by incubating at 70 °C overnight, and the resulting solution was freeze-dried (0.9 g). This solution was designated as fraction F60.

F60 (10 mg) was dissolved in 10 mL of deionized water by magnetic stirring at 70 °C overnight, followed by 24 h of dialysis (MWCO 6,000-8,000 Da) against 4 L of 0.1 M pyridine hydrochloride, then another 24 h of dialysis against 4 L of deionized water, and freeze-dried[10]. The resulting pyridinium salt of the sulfated galactan was subjected to solvolytic desulfation by heating in 10 mL of a DMSO/methanol mixture (9:1, v/v) at 80 °C for 6 h with magnetic stirring, followed by extensive dialysis (MWCO 6,000-8,000 Da) against deionized water[7]. The sample was permethylated using 1.2 mL of methyl iodide in 2 mL of DMSO in the presence of approximately 200 mg of NaOH powder[5]. An aliquot (2 mg) of the permethylated product was subjected to reductive hydrolysis and acetylation to generate partially methylated alditol acetates (PMAAs) [11]. In another experiment, F60 was permethylated, desulfated, and converted into PMAAs in the same manner, except that the desulfation treatment lasted for 16 h instead of 6 h. In a separate experiment, F60 without desulfation treatment was changed into its triethylammonium salt form by dialysis again 0.1 M triethylamine hydrochloride, permethylated, and then converted into C-1 deuterium-labeled PMAAs by 2 M TFA hydrolysis, $NaBD_4$ reduction, and acetylation, and also into PMAAs without deuterium labeling by reductive hydrolysis and acetylation[11]. PMAA derivatives were tested on an Agilent 7890A-5977B GC-MS system (Agilent Technologies, CA, USA) equipped with a Supelco SP-2380 column (100 m × 0.25 mm × 0.2 µm; Sigma-Aldrich, MA, USA), with oven temperature programmed to start at 100 °C (hold 1 min), followed by increases of 15 °C/min to 200 °C, and then 1 °C/min to 250 °C (hold 20 min). Inlet temperature was 250°C, and the constant column helium flow rate was 1.2 mL/min. The PMAAs were identified based on their EI-MS ion fragmentation patterns[8]. Relative molar linkage compositions of the PMAAs were estimated from the total ion current (TIC) chromatogram, based on the principle that the quantity of a PMAA is proportional to the ratio of its TIC peak area to its molecular mass[12]. For each sample, two separate experiments were conducted.

Supplementary Method 2

*Bx*MAG$_{BOV}$, *Bx*Car5$_{BOV}$, *Bx*Car17$_{BOV}$, control strain *B. xylanisolvens* *Bx*XB1A$_{HOM}$, and marine environment bacterium sharing the highest homology with carrageenan PUL CAZymes including *R. Alistipes* MAGs: FM_12_G117_V, FM_17_G117_V, FM_15_G124_V, *Wenyingzhuangia aestuarii* (GCF_011927765.1), *Lutibacter citreus* (GCF_003260195.1), *Labilibacter marinus*

(GCF_001659685.2), *Flavobacterium* sp. 7A (GCF_025960985.1), *Saccharicrinis aurantiacus* (GCF_947489045.1), and *Saccharicrinis fermentans* (GCF_000517085.1) were annotated with prodigal (v2.6.3) to obtain GFF files. *R. Alistipes* MAGs were chosen based on their synteny with CarPULs. GC usage was calculated as a % for each gene and GraphPad Prism was used to calculate means and deviation, and Brown-Forsythe and Welch ANOVAs. Games-Howell's comparisons was done between carrageenan PULs and genomes to compare differences between GC means. Asterisks denote P value cutoff's (ns: $P > 0.05$; *: $P \leq 0.05$; **: $P \leq 0.01$; ***: $P \leq 0.001$; ****: $P \leq 0.0001$).

Supplemental References

1. Barnett, D.J.M. microViz: an R package for microbiome data visualization and statistics. *Journal of Open Source Software* **6**, 3201 (2021).
2. R Core Team, R. R: A language and environment for statistical computing. (2013).
3. Yariv, B. et al. Using evolutionary data to make sense of macromolecules with a "face-lifted" ConSurf. *Protein Sci* **32**, e4582 (2023).
4. Despres, J. et al. Xylan degradation by the human gut Bacteroides xylanisolvens XB1A(T) involves two distinct gene clusters that are linked at the transcriptional level. *BMC Genomics* **17**, 326 (2016).
5. Bajwa, B. et al. Characterization of Unfractionated Polysaccharides in Brown Seaweed by Methylation-GC-MS-Based Linkage Analysis. *Marine Drugs* **22**, 464 (2024).
6. Stevenson, T.T. & Furneaux, R.H. Chemical methods for the analysis of sulphated galactans from red algae. *Carbohydrate Research* **210**, 277-298 (1991).
7. Nagasawa, K., Inoue, Y. & Kamata, T. Solvolytic desulfation of glycosaminoglycuronan sulfates with dimethyl sulfoxide containing water or methanol. *Carbohydrate Research* **58**, 47-55 (1977).
8. Carpita, N.C. & Shea, E.M. in Analysis of Carbohydrates by GLC and MS 157-216 (CRC press, 2021).
9. Xing, X. et al. Study on Dendrobium officinale O-acetyl-glucomannan (Dendronan®): Part I. Extraction, purification, and partial structural characterization. *Bioactive Carbohydrates and Dietary Fibre* **4**, 74-83 (2014).
10. Robb, C.S. et al. Metabolism of a hybrid algal galactan by members of the human gut microbiome. *Nature Chemical Biology* **18**, 501-510 (2022).
11. Bajwa, B., Xing, X., Terry, S.A., Gruninger, R.J. & Abbott, D.W. Methylation-GC-MS/FID-Based Glycosidic Linkage Analysis of Unfractionated Polysaccharides in Red Seaweeds. *Mar Drugs* **22** (2024).
12. Pettolino, F.A., Walsh, C., Fincher, G.B. & Bacic, A. Determining the polysaccharide composition of plant cell walls. *Nature Protocols* **7**, 1590-1607 (2012).
