# Supplementary material for: Distribution of microbial carrageenan foraging pathways reveals a widespread latent trait within the ruminant intestinal microbiome
Source: Nat Commun. 2026 May 12;17:4237. doi: 10.1038/s41467-026-70776-7 (PMC13168452; doi:10.1038/s41467-026-70776-7)
Supplement: Supplementary file 2 — Descriptions of Additional Supplementary Files [file 41467_2026_70776_MOESM2_ESM.pdf]

Description of supplementary files.

Supplementary Data 1: Statistics and GTDB-tk taxonomy for MAGs generated in this study (completeness > 70% and contamination > 10%).

Supplementary Data 2: Bovine metagenomics reads (this study) blasted against CarPUL genes.

Supplementary Data 3: Log_2 transformed metaproteomic data.

Supplementary Data 4: Bovine *Bx*MAG CarPUL genes blasted against other bovine *Bx*CarPULs.

Supplementary Data 5: Ruminant *Bx*CarPUL genes blasted against Human CarPULs.

Supplementary Data 6: Ruminant read sets blasted against Bovine CarPULs genes.

Supplementary Data 7: All ruminant fecal and rumen metagenomic assemblies.

Supplementary Data 8: *Mj*SM blast hits against CarPUL genes.

Supplementary Data 9: *Rikenellaceae* MAGs blast hits against CarPUL genes.

Supplementary Data 10: All zoo animals used within this study.

Supplementary Data 11: GC value statistics for all genomes compared.
