# Supplementary material for: Distribution of microbial carrageenan foraging pathways reveals a widespread latent trait within the ruminant intestinal microbiome
Source: Nat Commun. 2026 May 12;17:4237. doi: 10.1038/s41467-026-70776-7 (PMC13168452; doi:10.1038/s41467-026-70776-7)
Supplement: Supplementary file 12 — Supplementary Data 10 [file 41467_2026_70776_MOESM12_ESM.pdf]

Wetaskiwin Coop Assoc. LTD.
Stored Formula Report
Plant : 108 - WETASKIWIN - FP Pricing
Plant: 108 - WETASKIWIN - FP

Formula Code	Description	Species Code	Batch Weight	Date Stored	Ver
M800710	Calgary Zoo WINTER Herbivore	32	1000.00	17/02/2015	2

Code	Ingredient Name	Amount
BPPG	Ground Beet Pulp	401.23
DAF	ALF-DEHY-17.5	247.50
LINPRO	Linpro	95.00
WPP	MILLRUN	92.50
WDDG	Wheat Dist. Grain 50:50	50.00
OMEGAFLX	Milled Flax-steel cut	35.50
OFLAXOIL	ORGANIC FLAX OIL	30.00
RPS	Canola Meal	30.00
SLT	SALT	9.68
LIM	LIMESTONE	3.00
RVE	VIT E-50% ADS	1.48
CAP	DICAL PHOS-21%	1.00
MGO	MAG OX-56%	1.00
999	Selenium 1000 mg/kg (	0.84
111	TM SUL-Pak	0.68
333	B VIT PAK - P	0.20
222	ADE VIT PAK-30 Natur	0.17
334466	Sheep TM Micro Lt........	0.15
D3BLEND	Vit D 10,000 IU/kg	0.08

No.	Nutrient Name	Units	Actual
2	Protein	%	14.9507
3	Fat	%	7.8097
23	linoleic acid	%	2.9948
24	linolenic acid	%	0.6042
41	TDN-ruminant	%	72.4463
44	NE maint	mcal/kg	1.6784
50	ME poultry	kcal/kg	2,390.3750
56	ME swine	kcal/kg	2,610.8620
70	calcium	%	0.8315
71	phosphorus	%	0.3487
72	av phosphorus	%	0.1912
78	magnesium	%	0.3316

Wetaskiwin Coop Assoc. LTD.
Stored Formula Report
Plant : 108 - WETASKIWIN - FP Pricing
Plant: 108 - WETASKIWIN - FP

No.	Nutrient Name	Units	Actual
79	potassium	%	1.1510
81	sodium	%	0.5003
82	sulfur	%	0.1545
90	cobalt	mg/kg	0.7350
91	copper	mg/kg	17.0182
93	iodine	mg/kg	2.2875
94	iron	mg/kg	158.2434
95	manganese	mg/kg	135.8523
98	zinc	mg/kg	121.0324
110	selenium added	mg/kg	0.8400
116	vit A	KIU/kg	5.1000
117	vit D3	KIU/kg	1.3100
118	vit E	IU/kg	754.6800
119	vit K	mg/kg	2.0000
120	menadione	mg/kg	2.0000
122	biotin	mg/kg	0.3374
123	choline	mg/kg	1,025.6120
124	folic acid	mg/kg	1.9742
125	niacin	mg/kg	53.7980
126	pantothenic acid	mg/kg	16.3615
127	pyridoxine	mg/kg	4.8580
128	riboflavin	mg/kg	9.4146
129	thiamine	mg/kg	3.8966
130	vit B12	mcg/kg	16.0027
134	vit A added	KIU/kg	5.1000
135	vit D3 added	KIU/kg	1.3100
136	vit E added	IU/kg	754.5000
137	vit K added	mg/kg	2.0000
138	menadione added	mg/kg	2.0000
140	biotin added	mg/kg	0.2400
142	folic acid added	mg/kg	0.9000
143	niacin added	mg/kg	24.0000
144	pantothenic acid ad	mg/kg	7.0000
145	pyridoxine added	mg/kg	2.2000
146	riboflavin added	mg/kg	5.6000
147	thiamine added	mg/kg	1.3000
148	vit B12 added	mcg/kg	16.0000

DIET SHEET - Alpaca

calgaryzoo

**Common Name:**	Domestic Alpaca
**Scientific Name:**	*Lama pacos domestic*
**Animal Name (s):**	Latte (Fawn), Chai (white), Pekoe (brown)
**Accession Number:**	108955,109046/7
**Sex:**	F, F, F
**DOB:**	15-Jul-10/6-Aug-12/11-Aug-12
**Target BW Range (kg):**	72, 95, 95
**Target Calories (kcal):**	1.5-2.5%BW, 70:30 F:C
**Calories Provided:**	1.81 (2.8% BW), 80:20 F:C
**Avg. Intake (asfed)**	Intake not measured

Diet: Alpaca
Section: South America

Standard diet for : 1
Total same species in Enclosure: 0.3

STANDARD DIET FOR: 1 ANIMAL **DATE: 1-Sep-23**

Day							Food Type	Amount	Notes
M	T	W	R	F	Sa	Su	Summer Herbivore Pellets*	315 g	1 2/3 cup, Flash with water, sprinkle with suppl.
M	T	W	R	F	Sa	Su	Alpaca Mineral Supplement	30 g	balanced to 1.5ppm Se
M	T	W	R	F	Sa	Su	Ranch Mixed Hay**	1 flake	1.5kg/flake. Up to 4 flakes for the group Mixed hay, 10-20% alfalfa (10% CP)

NOVEMEMBER 2023 SWITCH TO MAZURI ALPACA MAINTENANCE

Notes: Diet is per animal
*Pellet consumption tends to fluctuate, refer to whiteboard in cedar barn kitchen for current amounts.
Pellets are offered in the am as a shifting incentive. Any pellets used for alpaca BTS should come out of daily rations.
One Alpaca (Pekoe) has a history of choking episodes, therefore always ensure pellets are moistened.

**When transitioning to a new mixed hay, always do this slowly over 12 - 14 days.

FEED AS INDICATED. DO NOT ALTER DIET. IF CHANGE IS REQUIRED PROVIDE DETAILS IN DIET CHANGE REQUEST.

European Wild Boar, Female

**Common Name:**	Wild Boar
**Scientific Name:**	*Sus scrofa scrofa*
**Animal Name (s):**	Fern, Poppy
**Accession Number:**	109140/109141
**Sex:**	F/F
**DOB:**	3-Apr-13/15-Apr-13
**Target BW Range (kg):**	160
**Target Calories:**	2XME
**Calories Provided:**	
**Avg. Intake (asfed)**	2.3, 1.4% BW

Diet: Adult Female
Section: Asia

Standard diet for : 1
Total same species in Enclosure: 1.2

STANDARD DIET FOR: **1** **ANIMAL** **DATE:** **4-Aug-22**

Day							Food Type	Amount	Notes
M	T	W	R	F	Sa	Su	Calgary Zoo Herbivore Cubes	1500 g	
M	T	W	R	F	Sa	Su	Enrichment Fruit	150 g	40% apple, remove large pits
M	T	W	R	F	Sa	Su	Enrichment vegetables	400 g	50% Yam
M	T	W	R	F	Sa	Su	Yam	250 g	
M		W		F			Greenie Dental Sticks - Large*	1 stick	

Preference list:
NO: Peppers of any color, NO carrots, NO celery
NO: turnip. NO cabbage, NO eggplant
NO: Orange, lemon, lime, or pinneapple

Notes: Diet is per individual. Monitor weights carefully, animals are prone to obesity and need to lose weight slowly.

FEED AS INDICATED. DO NOT ALTER DIET. IF CHANGE IS REQUIRED PROVIDE DETAILS IN DIET CHANGE REQUEST.

Recent changes:

*July-2022 begin trialing Greenie Dental Care 5" sticks for Large dog breeds to help with the chalky tartar build up vets noticed on their cheek teeth. Will reasses at next anesthetic event as these teeth are pretty deep in the mouth or in 6 months time (Jan 2023).
* Sept 26, 2023 - I wanted to note that Fern’s teeth looked great during her immobilization today with regards to there being minimal gingivitis or tartar/calculus build up. Hopefully the greenies continue to help - ST

Changes as of 12-July-2022
Started offering dental sticks for dogs (4 inch, large dog breed). Barb did not want to try XL size.
2-3x per week, and will reasses at next anesthesia event because the teeth are back behind cheeks,

and hard to see without a knockdown
Barb and team will monitor to ensure they are chewed with their cheek teeth for at least 60 seconds or else may be poinless.

Changes as of Spring 2022

Due to HPAI strain, boars were kept inside, and received cracked corn to keep them occupied they all gained weight after this based on vet exam.
Also vets determined a lot of tartar build up from anesthesia event, trial dental sticks for dogs

European Wild Boar, Male

**Common Name:**	Wild Boar
**Scientific Name:**	*sus scrofa*
**Animal Name (s):**	Wilbur
**Accession Number:**	109139
**Sex:**	M
**DOB:**	21-Mar-12
**Target BW Range (kg):**	200
**Target Calories:**	2XME
**Calories Provided:**	
**Avg. Intake (asfed)**	2.95, 1.5% BW

Diet: Adult Male
Section: Asia

Standard diet for : 1
Total same species in Enclosure: 1.2

STANDARD DIET FOR: **1** **ANIMAL** **DATE:** **4-Aug-22**

Day							Food Type	Amount	Notes
M	T	W	R	F	Sa	Su	Calgary Zoo Herbivore Cubes	1700 g	
M	T	W	R	F	Sa	Su	Enrichment Fruit	350 g	40% apple, remove large pits
M	T	W	R	F	Sa	Su	Enrichment vegetables	500 g	50% Yam
M	T	W	R	F	Sa	Su	Mixed hay	400 g	
	T		R		Sa		Walnut or Brazil Nut	2 nuts	For dental health
M		W		F			Greenie Dental Sticks - Large*	1 stick	

Preference list:
NO: Peppers of any color, NO carrots, NO celery
NO: turnip. NO cabbage, NO eggplant
NO: Orange, lemon, lime, or pinneapple

Notes: Diet is per individual. Monitor weights carefully, animals are prone to obesity and need to lose weight slowly.

FEED AS INDICATED. DO NOT ALTER DIET. IF CHANGE IS REQUIRED PROVIDE DETAILS IN DIET CHANGE REQUEST.

*July-2022 begin trialing Greenie Dental Care 5" sticks for Large dog breeds to help with the chalky tartar build up vets noticed on their cheek teeth. Will reasses at next anesthetic event as these teeth are pretty deep in the mouth or in 6 months time (Jan 2023).
* Sept 26, 2023 - I wanted to note that Fern’s teeth looked great during her immobilization today with regards to there being minimal gingivitis or tartar/calculus build up. Hopefully the greenies continue to help - ST

Camel, Bactrian

calgaryzoo

Common Name:	Bactrian camel
Scientific Name:	*Camelus bactrianus*
Animal Name (s):	Zsa-Zsa
Accession Number:	107222
Sex:	F
DOB:	23-Jun-05
Target BW Range:	750 (F)
Target Calories:	1.5% BW
Calories Provided:	1.12-1.3% BW
Avg. Intake (asfed)	

Diet: Maintenance
Section: Asia

Standard diet for : 1
Total same species in Enclosure: 0.1

STANDARD DIET FOR: **1** **ANIMAL** **DATE:** **17-Sep-23**

Day							Food Type	Amount	Notes
M	T	W	R	F	Sa	Su	Herbivore cubes (Zsa-Zsa)	1500 g	1 scoop
M	T	W	R	F	Sa	Su	Mixed hay (10-20% alfalfa, 10-13% CP)	5-6 flakes	1.5kg/flake
	T				Sa		Carrots	125 g each	
			R				Apples	75 g each	
M	T	W	R	F	Sa	Su	Blue Salt Block	free choice	
M	T	W	R	F	Sa	Su	Browse	as available	low priority species
M	T	W	R	F	Sa	Su	Herbivore cubes	200 g each	1/2 AM 1/2 PM
									enrichment, meds

Notes: Diet is per animal.
Low quality hay is sufficient, if too high in protein or alfalfa content, transition over 12-15 days, and may reduce amount offered. Assess BCS 2x per year.

FEED AS INDICATED. DO NOT ALTER DIET. IF CHANGE IS REQUIRED PROVIDE DETAILS IN DIET CHANGE REQUEST.

DIET SHEET - Caribou

**Common Name:**	American Woodland Caribou
**Scientific Name:**	*Rangifer tarandus caribou*
**Animal Name (s):**	Vanilla, Bean (Mica), Primrose, Avens
**Accession Number:**	109193, 111023, 111327, X
**Sex:**	F, F, F, F
**DOB:**	22-May-14, 29-Jun-20, 16-Jun-21, X
**Target BW Range (kg):**	125 (F), 150 (M)
**Target Calories (kcal/d):**	1.5-2.5% BW
**Calories Provided (kcal/d):**	1.5-2.5% BW
**As fed, DM (kg/d), (%BW):**	F (1.9-3.1kg/d), M (2.25-3.75kg/d)

**Diet:**	Caribou
**Section:**	Canadian Wilds
**Standard diet for :**	1
**Total same species in Enclosure:**	0.4

STANDARD DIET FOR: 1 ANIMAL **DATE: 1-Sep-23**

Day	Food Type	Amount	Notes
M T W R F Sa Su	Winter Herbivore Pellets*	1.5-2 rations per female	1 ration = 1500g
M T W R F Sa Su	Winter Herbivore Pellets*	2-3 rations per male	1 ration = 1500g
M T W R F Sa Su	Alfalfa hay	free choice round bale or 2 flakes/d	
M T W R F Sa Su	Cobalt blue salt lick	free choice	2kg size
M T W R F Sa Su	Lichen	as available	
M T W R F Sa Su	Approved Browse	free choice	Daily, 1st priority species 750g/d minimum 5% of forage
**LACTATING/NURSING ANIMALS**			
M T W R F Sa Su	Calf Manna Pro	200 g	Supplement to nursing animals
M T W R F Sa Su	Winter Herbivore Pellets	1.5-2x current ration	

Notes: Diet is per animal.

* Winter Herbivore Pellet fed year round, 1 ration = 1500g. When group fed, adjust up or down by one ration as needed. Only make 1 change per week, record in daily records.

When fed individually, increase or decrease by (10%) 150g as needed. Only make one change per week.

This species must be offered fresh or forzen browse daily

FEED AS INDICATED. DO NOT ALTER DIET. IF CHANGE IS REQUIRED PROVIDE DETAILS IN DIET CHANGE REQUEST.

Giraffe, Female

**Common Name:**	Giraffe
**Scientific Name:**	*Giraffa camelopardalis*
**Animal Name (s):**	Moshi, Emara
**Accession Number:**	109513, 109615
**Sex:**	F
**DOB:**	Oct. 2015, May 2011
**Target BW Range:**	850
**Target Calories:**	2% BW
**Calories Provided:**	2 %BW
**Avg. Intake (asfed, kg)**	17

**Diet:**	Maintenance
**Section:**	Savannah
**Standard diet for :**	1
**Total same species in Enclosure:**	1.2

STANDARD DIET FOR: 1 ANIMAL **DATE: 15-Oct-22**

Day							Food Type	Amount	Notes
M	T	W	R	F	S	Su	Winter Herbivore Pellets	3200 g	Moshi - 2900g (23-Jan-2021)
M	T	W	R	F	S	Su	Alfalfa Hay	8000 g	
M	T	W	R	F	S	Su	Vegetable Variety	200 g	
M	T	W	R	F	S	Su	Fruit Variety	50 g	
M	T	W	R	F	S	Su	Cobalt Blue Salt Block	free choice	
M		W		F			Primate Biscuits	1000 g	Training, hoof trims, etc
						Su	Carrots, Lettuce	1000 g	Zoo School, BTS, 1-4x per month
M	T	W	R	F	S	Su	Browse (Fresh, Frozen, or Silage)	5000 g	1st priority species<br>5% of forage, minimum requirement

Notes: Diet is per individual.
Winter Herbivore Pellets all year round.
Extra bananas reserved for medicating, not included in diet
Recommended 16-20% CP, 70:30 F:C

FEED AS INDICATED. DO NOT ALTER DIET. IF CHANGE IS REQUIRED PROVIDE DETAILS IN DIET CHANGE REQUEST.

Giraffe, Male

**Common Name:**	Giraffe
**Scientific Name:**	*Giraffa camelopardalis*
**Animal Name (s):**	Nabo
**Accession Number:**	109025
**Sex:**	M
**DOB:**	1-Jan-10
**Target BW Range (kg):**	1150
**Target Calories:**	2% BW
**Calories Provided:**	2 %BW
**Avg. Intake (asfed, kg)**	22.7

**Diet:**	Maintenance
**Section:**	Savannah
**Standard diet for :**	1
**Total same species in Enclosure:**	1.2

STANDARD DIET FOR: **1** **ANIMAL** **DATE:** **6-Aug-22**

Day							Food Type	Amount	Notes
M	T	W	R	F	S	Su	Winter Herbivore Pellets	3400 g	
M	T	W	R	F	S	Su	Alfalfa Hay	12000 g	
M	T	W	R	F	S	Su	Vegetable Variety	200 g	
M	T	W	R	F	S	Su	Fruit Variety	50 g	
M	T	W	R	F	S	Su	Cobalt Blue Salt Block	free choice	
M		W		F			Primate Biscuits	1000 g	Training, hoof trims, etc
						Su	Carrots, Lettuce	1000 g	Zoo School, BTS, 1-4x per month
M	T	W	R	F	S	Su	Browse (Fresh, Frozen, or Silage)	6500 g	1st priority species<br>5% of forage, minimum requirement

Notes: Diet is per individual.
Winter Herbivore Pellets all year round.
Recommended 16-20% CP, 70:30 F:C

FEED AS INDICATED. DO NOT ALTER DIET. IF CHANGE IS REQUIRED PROVIDE DETAILS IN DIET CHANGE REQUEST.

Wilder Institute Calgary Zoo

Rocky Mountain Goat

**Common Name:**	Rocky Mountain Goat
**Scientific Name:**	*Oreamnos americanus*
**Animal Name (s):**	Shannon, Amanda, Yukon, Peyto, Pika, Suncup, Hara
**Accession Number:**	107724, 5, 109032, 110805, 6, 111113, 111486
**Sex:**	F/F/M/F/F/F/F
**DOB:**	23-May-07 (2), 29-May-12, 18-May-20, (2), 10-May-21, 10-May-22
**Target BW Range (kg):**	75-85
**Target Calories:**	2-3.5%BW, 60:40 F:C
**Calories Provided:**	
**Avg. Intake (asfed)**	2.0-3.5% BW

**Diet:**	Maintenance
**Section:**	Canadian Wilds
**Standard diet for :**	1
**Total same species in Enclosure:**	1.6

STANDARD DIET FOR: 1 ANIMAL **DATE: 3-Aug-22**

Day							Food Type	Amount	Notes
M	T	W	R	F	S	Su	Calgary Zoo Herbivore Pellets*	1100 g	7700 g for group maintenance**
M	T	W	R	F	S	Su	Mixed Hay (25% alfalfa)	2/3 flake	Grassy, soft, 10-20% alfalfa, 16-18%CP
M	T	W	R	F	S	Su	Cobalt Blue Salt Block	ad libitum	
					S		Romaine	0.25 hd	1.5hd for group
M	T	W	R	F	S	Su	Browse	as available	2nd priority species<br>2.5% of forage, 300g/d
							Edible Flowers	as available	seasonal, by donation

Notes: Diet is per animal.

*Summer herbivore pellet April to Oct. Transition over 12 days 25:75 (4 days), 50:50 (4 days), 75:25 (4 days), 100%

*Winter herbivore pellet Nov to Mar. Transition over 12 days 25:75 (4 days), 50:50 (4 days), 75:25 (4 days), 100%

Consult animal nutrition supervisor for current mixed hay components and recommended amounts.

Recommended, 25% Alfalfa, 16-18%CP, 90% DMB

Pasture available in summer, hay may be reduced based on appetite and body condition

**Adjust amount of herbivore pellets based on number of animals and stage of gestation (2x in 3rd trimester) or lactation (3x). Offer Foal Lac to goat kids.

Difficult to enrich with food, as they are very picky. Scent enrichment is recommended.

FEED AS INDICATED. DO NOT ALTER DIET. IF CHANGE IS REQUIRED PROVIDE DETAILS IN DIET CHANGE REQUEST.

Hippo, Adult

Wilder Institute Calgary Zoo

**Common Name:**	Hippopotamus
**Scientific Name:**	*Hippopotamus amphibius*
**Animal Name (s):**	Lobi, Sparky
**Accession Number:**	109024, 102964
**Sex:**	M, F
**DOB:**	31-Oct-06, 30-May-87
**Target BW Range (kg):**	1900, 1400 (est)
**Est. Pred. Equ. (kcal/d):**	40290, 32042
**Calories Provided (kcal/d):**	21577
**Intake asfed, kg, %BW:**	14.1, 0.85

Diet: Maintenance
Section: Savannah

Standard diet for : 1
Total same species in Enclosure: 1.1

STANDARD DIET FOR: 1 ANIMAL **DATE: 9-Feb-21**

Day							Food Type	Amount			Notes
M	T	W	R	F	Sa	Su	Herbivore Cubes	3000	g	each	
M	T	W	R	F	Sa	Su	Vitamin E (50%)	2	tsp.	each	sprinkle on top of cubes
M	T	W	R	F	Sa	Su	Biotin (2%)	1.5	tsp.	each	sprinkle on top of cubes
M	T	W	R	F	Sa	Su	Timothy hay	4	flakes	each	~ 6kg/day each
M	T	W	R	F	Sa	Su	Mixed Hay Alfalfa-grass hay	1.5	2" flake	each	~ 3kg/day each
M	T	W	R	F	Sa	Su	Romaine	1600	g	2 hd each	fed in pool at any time
M	T	W	R	F	Sa	Su	Fruit	300	g	1000g for 1.1, training/enrichment	
M	T	W	R	F	Sa	Su	Vegetables	200	g		

Notes: Calories calculated using Kleiber 2XBMR (LJ)
4-May-2022 - Lobi's diet reduced to Adult ration (3500g to 3000g herb cubes over 4 weeks)

FEED AS INDICATED. DO NOT ALTER DIET. IF CHANGE IS REQUIRED PROVIDE DETAILS IN DIET CHANGE REQUEST.

Markhor

Common Name:	Markhor
Scientific Name:	*Capra falconeri*
Animal Name (s):	Sproing, Cliff
Accession Number:	107234, 111490
Sex:	F, M
DOB:	12-May-05, 17-Aug-21
Target BW Range:	48 (F), growth (M)
Target Calories:	1.5-2.5% BW
Calories Provided:	
Avg. Intake (asfed)	1.8-4% BW

Diet: Maintenance

Section: ASIA

Standard diet for : 1

Total same species in Enclosure: 1.1

STANDARD DIET FOR: 1 **ANIMAL** **DATE:** 17-Sep-23

Day							Food Type	Amount	g	Group (1.1) Amount
M	T	W	R	F	Sa	Su	Winter Herbivore Pellet	1.5-3 cups	265-530	3-6 cups
M	T	W	R	F	Sa	Su	Wild Herbivore Plus	0.75-1.5 cups	125-250	1.5-3 cups
							2:1 ratio winter:WH+			
M	T	W	R	F	Sa	Su	Mixed Hay (>50% alfalfa)	1/4-1/2 flakes	375-750	1.5-2 flakes
	T				Sa		Romaine	1/4 hd	200	1/2 hd
		W		F		Su	Apple or Carrot	50 g		100 g
M	T	W	R	F	Sa	Su	Cobalt Blue Salt Block	free choice		
M	T	W	R	F	Sa	Su	Browse	as available		2nd priority browse species min 225g/day, 2.5% of forage

Notes: Diet is per animal. Group amount shown for 1.1.0

Hay and Pellet intake changes seasonally and is recorded daily on section, adjusted as needed.

Feed high quality hay (>50% alfalfa, CP>14%) over the winter (Nov to June), may reduce quality July to Oct (<50% alfalfa, CP 11-13%).

Remain on Winter Herb Pellet and WH+ year round

Produce is used for training and enrichment purposes, or medicating.

Breeding Season Jan (rut) - April, may reduce appetite and BCS.

Pregnant animals in 3rd trimester or lactating animals may receive upto 30% increase in pellet and hay components.

BCS assessed quartely (May/Jun, Aug/Sept, Nov/Dec, and Feb/Mar)

Offer boost at 10% of diet for thin/geriatic animals (75-150g/d).

FEED AS INDICATED. DO NOT ALTER DIET. IF CHANGE IS REQUIRED PROVIDE DETAILS IN DIET CHANGE REQUEST.

Mazuri®

EXOTIC ANIMAL NUTRITION

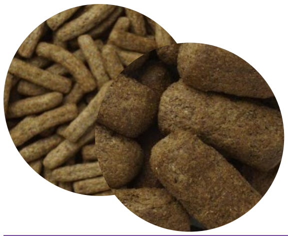

Leaf-Eater Primate Diets

Mazuri® Leaf-Eater Primate Diets are complete lifecycle diets specially formulated to meet the needs of leaf-eating primates which are thought to require a high-fiber diet such as lemurs, langurs, and howlers. This diet can be fed to other species of primates such as gorillas and orangutans, when a high-fiber diet is desired.

Features and Benefits

- **Designed to be fed with supplementation** – Allows for natural feeding behaviors by the addition of species-appropriate food items.
- **Meets NRC recommendations for all nutrients** – Except protein, sodium, and chloride when fed at 50% of the diet.
- **High fiber** – Contains multiple sources of soluble and insoluble fiber.
- **No added sucrose or fructose** – Helps maintain dental health and may be appropriate for sugar-sensitive primates.
- **No added wheat product** – May be appropriate for animals with wheat sensitivity.
- **Enriched with Vitamins E and vitamin C** – Supports animal wellness.
- **Contains flaxseed** – Source of Omega-3 fatty acids.

Product Form & Packaging

Extruded Particle | 25 lb. (11.33 kg) net weight paper sack

- **Catalog #0001472** | 1” x 2” Biscuit
- **Catalog #0001448** | 1/4” x 1” Mini biscuit

Guaranteed Analysis

Crude protein not less than	23.00%
Crude fat not less than	5.00%
Crude fiber not more than	14.00%
Moisture not more than	12.00%
Ash not more than	9.00%

Ingredients

Dehulled Soybean Meal, Ground Soybean Hulls, Ground Corn, Corn Gluten Meal, Ground Oats, Dried Plain Beet Pulp, Dried Apple Pomace, Soybean Oil, Dehydrated Alfalfa Meal, Dicalcium Phosphate, Calcium Carbonate, Ground Flaxseed, Brewers Dried Yeast, Salt, L-Ascorbyl-2-Polyphosphate (Vitamin C), DL-Methionine, Pyridoxine Hydrochloride, Choline Chloride, Folic Acid, Vitamin A Acetate, Cholecalciferol (Vitamin D3), d-Alpha Tocopheryl Acetate (Vitamin E), Calcium Pantothenate, Ferrous Sulfate, Menadione Sodium Bisulfite Complex (Vitamin K), Preserved with Mixed Tocopherols, Manganous Oxide, Zinc Oxide, Rosemary Extract, Ferrous Carbonate, Nicotinic Acid, Citric Acid (a Preservative), Thiamine Mononitrate, Vitamin B12 Supplement, Riboflavin Supplement, Copper Sulfate, Zinc Sulfate, Calcium Iodate, Cobalt Carbonate, Sodium Selenite, Biotin.

Feeding Directions

- Mazuri® Primate Diets are designed to be an essential part of a total primate feeding system and may be used in conjunction with all other Mazuri® primate products.
- Primates generally consume 2% to 4% of their body weight in food each day on a dry matter basis (i.e., a 50 lb. animal will eat 1 to 2 kg of food per day, on a dry matter basis).
  - Mazuri® primate diets can be supplemented with fresh vegetables and fruit if this is desired to provide variety in the diet, as long as the dry matter of these items does not exceed 50% of the dry matter consumption. Using typical produce and non-toxic browse, a feeding program on an “as fed” basis might consist of 60% produce/browse and 40% Mazuri® primate products (i.e., a diet may be 6 lb. of produce/browse plus 4 lb. of one or a mix of Mazuri® primate products).
- The amount of diet the animal requires will vary according to its age, size, life stage, health status, and activity of the animal as well as the environmental temperature.
  - Adjust daily feed intake based on the health status, body condition, and nutrient level desired to meet the needs of your primates.
  - Diet may be soaked to soften the biscuits for neonates or animals that have difficulty chewing.
- Primates require ascorbic acid (vitamin C) in their daily diet. Mazuri® Primate Diets contain a stabilized form of vitamin C that is stable under appropriate storage conditions for at least 1 year from the date of manufacturing printed on the bag.
- Always provide plenty of fresh, clean water. Thoroughly wash feed and water bowls on a regular basis. It is always good practice to wash hands thoroughly after feeding and/or handling animals.
- This diet is not for human consumption.

Storage Conditions

For best results, reseal the bag between uses or store contents of open paper sack in container with sealing lid. Store in a cool (75°F/24°C or colder), dry (approximately 50% RH) location free from rodents and insects. Do not offer moldy or insect-infested feed to animals as it may result in illness, performance loss or death. Freezing will not harm the diet and may extend freshness. Use within 1 year of bag manufacturing or "Best if Used By" date.

Mazuri® is a registered trademark of PMI Nutrition International, LLC.

Moose

calgaryzoo

**Common Name:**	Moose
**Scientific Name:**	*Alces americanus*
**Animal Name (s):**	Maple, Aspen
**Accession Number:**	109978, 110242
**Sex:**	F/F
**DOB:**	26-May-18, 18-May-07
**Target BW Range (kg):**	250-300
**Target Calories:**	3-4% BW, 15-20%CP
**Calories Provided:**	3.4% BW, 60:40 F:C
**Avg. Intake (asfed)**	seasonal

Diet: Maintenance
Section: Canadian Wilds

Standard diet for : 1
Total same species in Enclosure: 0.2

STANDARD DIET FOR: **1** **ANIMAL** **DATE:** **1-Jul-22**

Day							Food Type	Amount	Notes
M	T	W	R	F	Sa	Su	Mazuri Moose Maintenance	3600 g	between 2500 and 4500g*
M	T	W	R	F	Sa	Su	alfalfa hay, prime quality	250 g	seasonal intake
M	T	W	R	F	Sa	Su	Cobalt Blue Salt Block	ad libitum	
M	T	W	R	F	Sa	Su	Browse	as available	1st priority species<br>2.5kg/day min, 5% of forage

Notes: *Diet intake changes seasonally. Monitor intake and fecal scores daily.
Offer Moose Breeder to breeding animals or 50:50 (breeder: maintenance) to growing moose upto the age of 4, double intake during 3rd trimester and lactation.

FEED AS INDICATED. DO NOT ALTER DIET. IF CHANGE IS REQUIRED PROVIDE DETAILS IN DIET CHANGE REQUEST.

Musk Deer

**Common Name:**	Siberian Musk Deer
**Scientific Name:**	*M. moschiferus*
**Animal Name (s):**	Ozzy
**Accession Number:**	109476
**Sex:**	M
**DOB:**	12-Jun-13
**Target BW Range (kg):**	11-12
**Target Calories (kcal/d):**	891-1366
**Calories Provided:**	1047
**Avg. Intake (asfed, kg)**	0.56 (+ forage)

Diet: Maintenance
Section: Asia

Standard diet for : 1
Total same species in Enclosure: 1.0

STANDARD DIET FOR: 1 ANIMAL **DATE: 10-Oct-23**

Day							Food Type	Amount	Notes
M	T	W	R	F	S	Su	Mazuri Mini Leaf-Eater	137 g	1, 1/4 cup
M	T	W	R	F	S	Su	Winter Herbivore Pellets	132 g	3/4 cup
M	T	W	R	F	S	Su	Yam	60 g	chopped 2mmx2mmx2mm
M	T	W	R	F	S	Su	Alfalfa Hay	1/6 flake	2 large handfuls (250g), mostly leaf
M	T	W	R	F	S	Su	Cobalt Blue Salt block	1 block	available all times
M	T	W	R	F	S	Su	Browse	250 g	2nd priority species<br>35g/d, 2.5% forage

Notes: Diet is per animal per day.
Diet accounts for seasonal variation in intake and pests.

FEED AS INDICATED. DO NOT ALTER DIET. IF CHANGE IS REQUIRED PROVIDE DETAILS IN DIET CHANGE REQUEST.

Sheep, Bighorn

calgaryzoo

**Common Name:**	Bighorn Sheep
**Scientific Name:**	*Ovis canadensis*
**Animal Name (s):**	Callie, Bill Williams, Cheyenne, June, Cash
**Accession Number:**	107661,109708/9,107962,110144
**Sex:**	F, M, F, F, F
**DOB:**	15-May-06, 3-May-17, 3-May-17, 4-Jun-18, 18-Jul-18
**Target BW Range (kg):**	35-90
**Target Calories:**	2-3.5%BW, 60:40 F:C
**Calories Provided:**	
**Avg. Intake (asfed)**	

Diet: Maintenance

Section: CW

Standard diet for : 1

Total same species in Enclosure: 1.4

STANDARD DIET FOR: 1 ANIMAL **DATE: 3-Aug-22**

Day							Food Type	Amount	Notes
M	T	W	R	F	S	Su	Calgary Zoo Herbivore Pellets*	750 g	3750g/group
M	T	W	R	F	S	Su	Mixed Hay (25% alfalfa)	3/5 flake	3 flakes/group, 4.5kg/day
M	T	W	R	F	S	Su	Cobalt Blue Salt Block	ad libitum	
					S		Romaine	0.25 hd	1.5hd for group
M	T	W	R	F	S	Su	Browse	as available	2nd priority species<br>250-500g/day, 2.5% of forage
							Edible Flowers	as available	seasonal, by donation

Notes: Diet is per animal. This is a Copper sensitive species, highly susceptible to Cu toxicity.

*Summer herbivore pellet April to Oct. Transition over 12 days 25:75 (4 days), 50:50 (4 days), 75:25 (4 days), 100%

*Winter herbivore pellet Nov to Mar. Transition over 12 days 25:75 (4 days), 50:50 (4 days), 75:25 (4 days), 100%

Consult animal nutrition supervisor for current mixed hay components and recommended amounts.

Recommended, 25% Alfalfa, 15-18%CP, 90% DMB

Pasture available in summer., hay may be reduced based on appetite and body condition

Adjust amount of herbivore pellets based on number of animals and stage of gestation or lactation (currenlty not breeding, all animals are related)

Difficult to enrich with food, as they are very picky. Scent enrichment is recommended.

FEED AS INDICATED. DO NOT ALTER DIET. IF CHANGE IS REQUIRED PROVIDE DETAILS IN DIET CHANGE REQUEST.
