# Supplementary material for: Distribution of microbial carrageenan foraging pathways reveals a widespread latent trait within the ruminant intestinal microbiome
Source: Nat Commun. 2026 May 12;17:4237. doi: 10.1038/s41467-026-70776-7 (PMC13168452; doi:10.1038/s41467-026-70776-7)
Supplement: Supplementary file 14 — Reporting Summary [file 41467_2026_70776_MOESM14_ESM.pdf]

nature portfolio

Corresponding author(s): D. Wade Abbott

Last updated by author(s): Mar 28, 2025

Reporting Summary

Nature Portfolio wishes to improve the reproducibility of the work that we publish. This form provides structure for consistency and transparency in reporting. For further information on Nature Portfolio policies, see our Editorial Policies and the Editorial Policy Checklist.

Statistics

For all statistical analyses, confirm that the following items are present in the figure legend, table legend, main text, or Methods section.

n/a	Confirmed	
☐	☒	The exact sample size (*n*) for each experimental group/condition, given as a discrete number and unit of measurement
☐	☒	A statement on whether measurements were taken from distinct samples or whether the same sample was measured repeatedly
☐	☒	The statistical test(s) used AND whether they are one- or two-sided<br>*Only common tests should be described solely by name; describe more complex techniques in the Methods section.*
☐	☒	A description of all covariates tested
☐	☒	A description of any assumptions or corrections, such as tests of normality and adjustment for multiple comparisons
☐	☒	A full description of the statistical parameters including central tendency (e.g. means) or other basic estimates (e.g. regression coefficient) AND variation (e.g. standard deviation) or associated estimates of uncertainty (e.g. confidence intervals)
☐	☒	For null hypothesis testing, the test statistic (e.g. *F*, *t*, *r*) with confidence intervals, effect sizes, degrees of freedom and *P* value noted<br>*Give P values as exact values whenever suitable.*
☒	☐	For Bayesian analysis, information on the choice of priors and Markov chain Monte Carlo settings
☒	☐	For hierarchical and complex designs, identification of the appropriate level for tests and full reporting of outcomes
☒	☐	Estimates of effect sizes (e.g. Cohen's *d*, Pearson's *r*), indicating how they were calculated

Our web collection on statistics for biologists contains articles on many of the points above.

Software and code

Policy information about availability of computer code

Data collection	metagenomic read sets were collected via NCBI-vdb (v3.1.0)
Data analysis	Kraken2 (v2.1.2 SILVA NR99 database) was used for 16S rRNA sequencing annotation. Kraken-biome was used to create a biome file for phyloseq (v1.48.0). Statistical analysis was done using the micoViz R package (v0.12.4), dplyr (v1.1.4), and ggplot2 (v3.5.1). Raw reads were quality trimmed using Trimmomatic. Shotgun metagenomic reads were assembled individually using MetaSPAdes (v3.13.0), and biological replicates together in a co-assembly using MEGAHIT (v1.1.3). Contigs were binned and refined using the MetaWRAP (v1.3.2) binning/refinement modules, with metaBAT2 (v2.12.1), MaxBin2 (v2.2.6), and CONCOCT (v1.1.0). Bins/MAGs between individual assemblies and co-assemblies were merged and de-replicated with dRep (v3.0.0). Bacterial isolate read sequences were assembled via Unicyler (v0.4.8). MAGs and ruminant isolates were taxonomically classified using GTDB-Tk (v2.3.2 - R202) and CheckM2 (v1.1.3), and were functionally annotated with DRAM (v1.3.4), prodigal (v2.6.3) and dbCAN3. MAGS/genomes were checked for quality using Quast (v5.0.2). MS raw data were analysed using FragPipe, powered by the proteomic search engine MSFragger along with Philosopher and IonQuant. Volcano plots were created using Perseus (v. 2.0.7.0). Diamond BLASTx (v2.1.8) was used for BLAST analysis and phylogenetic trees were built using SACCHARIS, OrthoFinder (v2.5.5), and iTOL. GraphPad Prism (v10.2.3) was used for statistical analysis of GC content. Initial phases for BxMAGBOV GH16A were determined by PHASER and a model of BxMAGBOV GH16A generated with AlphaFold 2. This initial model was manually corrected with COOT

For manuscripts utilizing custom algorithms or software that are central to the research but not yet described in published literature, software must be made available to editors and reviewers. We strongly encourage code deposition in a community repository (e.g. GitHub). See the Nature Portfolio guidelines for submitting code & software for further information.

Data

Policy information about availability of data

All manuscripts must include a data availability statement. This statement should provide the following information, where applicable:

- Accession codes, unique identifiers, or web links for publicly available datasets
- A description of any restrictions on data availability
- For clinical datasets or third party data, please ensure that the statement adheres to our policy

The mass spectrometry proteomics data have been deposited to the ProteomeXchange Consortium via the PRIDE partner repository with the dataset identifier PXD060679. All sequencing data have been deposited to the NCBI sequence read archive (SRA) under the project accession numbers PRJNA1227608. The functionally annotated MAGs and the MjSM protein database are available on Figshare (DOI: 10.6084/m9.figshare.28464551). The coordinates and Crystal structure factors for BxMAGBOV GH16_17A have been deposited in submitted to the Protein Data Bank under the identifier 9EFL. LC-ESI-MS data is deposited on GlycoPOST under the ID GPST000613. Previously published datasets used in this study as reference material are provided in the Source Data. Source data are associated with this manuscript. R scripts used for 16S analysis can be found on Zenodo (10.5281/zenodo.18775548).

Research involving human participants, their data, or biological material

Policy information about studies with human participants or human data. See also policy information about sex, gender (identity/presentation), and sexual orientation and race, ethnicity and racism.

Reporting on sex and gender	N/A
Reporting on race, ethnicity, or other socially relevant groupings	N/A
Population characteristics	N/A
Recruitment	N/A
Ethics oversight	N/A

Note that full information on the approval of the study protocol must also be provided in the manuscript.

Field-specific reporting

Please select the one below that is the best fit for your research. If you are not sure, read the appropriate sections before making your selection.

☒ Life sciences ☐ Behavioural & social sciences ☐ Ecological, evolutionary & environmental sciences

For a reference copy of the document with all sections, see nature.com/documents/nr-reporting-summary-flat.pdf

Life sciences study design

All studies must disclose on these points even when the disclosure is negative.

Sample size	two separate farm studies where conducted. one study with 4 cattle, another with 10 cattle. Single samples from zoo animals (n = 11) were collected.
Data exclusions	no data was excluded
Replication	All farm sampling experiments were completed with 4-5 biological replicates. Single samples from zoo animals were collected.
Randomization	Cattle were randomly assigned into treatment and control feed groups. Random zoo animals were collected.
Blinding	No studies were carried out blind, however biological replicates were used to prevent bias.

Reporting for specific materials, systems and methods

We require information from authors about some types of materials, experimental systems and methods used in many studies. Here, indicate whether each material, system or method listed is relevant to your study. If you are not sure if a list item applies to your research, read the appropriate section before selecting a response.

Materials & experimental systems	
n/a	Involved in the study
☒	☐ Antibodies
☒	☐ Eukaryotic cell lines
☒	☐ Palaeontology and archaeology
☐	☒ Animals and other organisms
☒	☐ Clinical data
☒	☐ Dual use research of concern
☒	☐ Plants

Methods	
n/a	Involved in the study
☒	☐ ChIP-seq
☒	☐ Flow cytometry
☒	☐ MRI-based neuroimaging

Animals and other research organisms

Policy information about studies involving animals; ARRIVE guidelines recommended for reporting animal research, and Sex and Gender in Research

Laboratory animals	This study did not contain laboratory animals.
Wild animals	This study did not include wild animals.
Reporting on sex	Sex was not considered in this study design. Sex was randomly assigned in the ad libitum trial, and steers were used in the silage experiment.
Field-collected samples	Samples were collected from two different pastures. +/- seaweed additives.
Ethics oversight	All procedures and protocols involving cattle were reviewed and approved by the Thompson Rivers University animal care committee (award #: 101948). with cattle cared for following the guidelines of the Canadian Council on Animal Care. Faecal collection from animals at the Wilder Institute/Calgary Zoo was approved by the Wilder Institute/Calgary Zoo Animal Welfare, Ethics, and Research Review Committee (Protocol 2023-06).

Note that full information on the approval of the study protocol must also be provided in the manuscript.

Plants

Seed stocks	N/A
Novel plant genotypes	N/A
Authentication	N/A
